# Supplementary material for: P-TRAP: a Panicle Trait Phenotyping tool
Source: BMC Plant Biol. 2013 Aug 29;13:122. doi: 10.1186/1471-2229-13-122 (PMC3848748; doi:10.1186/1471-2229-13-122)
Supplement: Additional file 7 — P-TRAP source code. The Netbeans project that contains the source code of the application. [file 1471-2229-13-122-S7.zip › javadoc.zip!/javadoc/org-basmati-workspace/org/basmati/workspace/StructureFinder.html]

StructureFinder (WorkSpace)


JavaScript is disabled on your browser.


- Package
- Class
- Tree
- Deprecated
- Index
- Help

*org.basmati.workspace 1.0*

- Prev Class
- Next Class

- Frames
- No Frames

- All Classes

- Summary:
- Nested |
- Field |
- Constr |
- Method

- Detail:
- Field |
- Constr |
- Method


org.basmati.workspace

## Class StructureFinder

- java.lang.Object
- - org.basmati.workspace.StructureFinder

- ---

    

  ```
  public abstract class StructureFinder
  extends java.lang.Object
  ```

  This class detects the structure of the panicle either in a single or
  multiple images.

  Author:
  :   faroq

- - ### Constructor Summary

    Constructors

    Constructor and Description

    `StructureFinder()`
  - ### Method Summary

    Methods

    | Modifier and Type | Method and Description |
    | --- | --- |
    | `static void` | `findStructure(org.openide.filesystems.FileObject file)` Find the structure in a single file. |
    | `static void` | `findStructure(org.openide.filesystems.FileObject[] files)` Find the structure of different files. |

    - ### Methods inherited from class java.lang.Object

      `clone, equals, finalize, getClass, hashCode, notify, notifyAll, toString, wait, wait, wait`

- - ### Constructor Detail


    - #### StructureFinder

      ```
      public StructureFinder()
      ```
  - ### Method Detail


    - #### findStructure

      ```
      public static void findStructure(org.openide.filesystems.FileObject file)
      ```

      Find the structure in a single file. The result will be stored in a
      result file.

      Parameters:
      :   `file` - A `FileObject` object.


    - #### findStructure

      ```
      public static void findStructure(org.openide.filesystems.FileObject[] files)
      ```

      Find the structure of different files.

      Parameters:
      :   `files` - an array of `FileObject`

      See Also:
      :   `findStructure(org.openide.filesystems.FileObject)`


- Package
- Class
- Tree
- Deprecated
- Index
- Help

*org.basmati.workspace 1.0*

- Prev Class
- Next Class

- Frames
- No Frames

- All Classes

- Summary:
- Nested |
- Field |
- Constr |
- Method

- Detail:
- Field |
- Constr |
- Method
